# Intratracheal IL-6 Protects against Lung Inflammation in Direct, but Not Indirect, Causes of Acute Lung Injury in Mice

**DOI:** 10.1371/journal.pone.0061405

**Published:** 2013-05-08

**Authors:** Rhea Bhargava, William Janssen, Christopher Altmann, Ana Andrés-Hernando, Kayo Okamura, R. William Vandivier, Nilesh Ahuja, Sarah Faubel

**Affiliations:** 1 Department of Medicine, Division of Renal Diseases and Hypertension, University of Colorado Denver, Aurora, Colorado, United States of America; 2 Division of Pulmonary Medicine, Department of Medicine, National Jewish Health, Denver, Colorado, United States of America; 3 Department of Medicine, Division of Pulmonary Medicine, University of Colorado Denver, Aurora, Colorado, United States of America; 4 Department of Medicine, Division of Renal Diseases and Hypertension, University of Colorado Denver, Aurora, Colorado, United States of America; 5 Department of Medicine Denver Veterans Affairs Medical Center, Denver, Colorado, United States of America; French National Centre for Scientific Research, France

## Abstract

**Introduction:**

Serum and bronchoalveolar fluid IL-6 are increased in patients with acute respiratory distress syndrome (ARDS) and predict prolonged mechanical ventilation and poor outcomes, although the role of intra-alveolar IL-6 in indirect lung injury is unknown. We investigated the role of endogenous and exogenous intra-alveolar IL-6 in AKI-mediated lung injury (indirect lung injury), intraperitoneal (IP) endotoxin administration (indirect lung injury) and, for comparison, intratracheal (IT) endotoxin administration (direct lung injury) with the hypothesis that IL-6 would exert a pro-inflammatory effect in these causes of acute lung inflammation.

**Methods:**

Bronchoalveolar cytokines (IL-6, CXCL1, TNF-α, IL-1β, and IL-10), BAL fluid neutrophils, lung inflammation (lung cytokines, MPO activity [a biochemical marker of neutrophil infiltration]), and serum cytokines were determined in adult male C57Bl/6 mice with no intervention or 4 hours after ischemic AKI (22 minutes of renal pedicle clamping), IP endotoxin (10 µg), or IT endotoxin (80 µg) with and without intratracheal (IT) IL-6 (25 ng or 200 ng) treatment.

**Results:**

Lung inflammation was similar after AKI, IP endotoxin, and IT endotoxin. BAL fluid IL-6 was markedly increased after IT endotoxin, and not increased after AKI or IP endotoxin. Unexpectedly, IT IL-6 exerted an *anti-inflammatory* effect in healthy mice characterized by reduced BAL fluid cytokines. IT IL-6 also exerted an anti-inflammatory effect in IT endotoxin characterized by reduced BAL fluid cytokines and lung inflammation; IT IL-6 had no effect on lung inflammation in AKI or IP endotoxin.

**Conclusion:**

IL-6 exerts an anti-inflammatory effect in direct lung injury from IT endotoxin, yet has no role in the pathogenesis or treatment of indirect lung injury from AKI or IP endotoxin. Since intra-alveolar inflammation is important in the pathogenesis of direct, but not indirect, causes of lung inflammation, IT anti-inflammatory treatments may have a role in direct, but not indirect, causes of ARDS.

## Introduction

Acute kidney injury (AKI) occurs in 20% of hospital admissions [1] and 30–50% of admissions to the intensive care unit (ICU) [2]. AKI confers an increased risk for respiratory complications [3] that include respiratory failure [4], [5], [6], prolonged mechanical ventilation [7], [8], [9], and prolonged weaning [9]. The cause of respiratory complications in patients with AKI is incompletely understood; however, we [10], [11] and others [12], [13], [14], [15], [16], [17], [18], [19] have shown that AKI in animals leads to lung inflammation and injury, characterized by lung neutrophil accumulation, increased lung capillary leak, and increased lung chemokine production [10], similar to that observed in other animal models of acute lung injury (ALI) such as sepsis [19].

IL-6 is a proinflammatory cytokine that plays a key role in the pathogenesis of AKI-mediated lung injury. In patients with AKI, serum IL-6 is increased and higher levels are associated with prolonged mechanical ventilation [7], [20] and increased mortality [21]. In mice, strategies to inhibit IL-6 reduced AKI-mediated lung injury [22]. Serum IL-6 is also increased in patients with ARDS and, like AKI, higher levels predict prolonged mechanical ventilation [23] and increased mortality [23], [24]. IL-6 is also increased in the bronchoalveolar lavage (BAL) fluid of patients with ARDS and high levels are associated with adverse outcomes [25], [26]; thus, IL-6 may contribute to lung injury via direct effects in the alveolar space.

The local effect of IL-6 in the alveolar space and upon alveolar macrophages in AKI-mediated lung injury is unknown. Data suggest that alveolar macrophages may indeed be important in the pathogenesis AKI-mediated lung injury as mice with alveolar macrophage depletion and AKI had reduced lung inflammation [27]. Therefore, in the present study, we sought to examine the role of intra-alveolar IL-6 in the pathogenesis of AKI-mediated lung injury and, for comparison, sepsis (IP endotoxin) and direct lung injury (IT endotoxin). AKI and sepsis are both causes of indirect lung injury. We hypothesized that intra-alveolar IL-6 would be a key proinflammatory mediator in all three causes of injury. Contrary to expectations, we found that intra-alveolar IL-6 exerted an anti-inflammatory effect when administered to healthy mice. Therefore, the effect of IT IL-6 to *prevent* lung injury was tested; interestingly, lung inflammation was reduced in only direct lung injury and was not affected in AKI-mediated lung injury or sepsis. The studies in this report highlight the key differences in the pathogenesis of three different causes of lung injury and indicate that indirect and direct causes of ALI have distant characteristics that may affect response to specific therapies.

## Methods

### Animals

8- to 10-wk-old male C57BL/6 mice (Jackson Laboratories, Bar Harbor, ME) weighing 20 to 25 grams were used. Mice were maintained on a standard diet, and water was freely available.

### Ethics Statement

All experiments were conducted with adherence to the National Institutes of Health Guide for the Care and Use of Laboratory Animals. The animal protocol was approved by the Animal Care and Use Committee of the University of Colorado, Denver.

### Surgical protocol for sham operation and ischemic AKI

For sham operation and ischemic AKI, mice were anesthetized with IP avertin (2,2,2-tribromoethanol; Aldrich, Milwaukee, WI), a midline incision was made, and the renal pedicles were identified. In the ischemic AKI group, both renal pedicles were clamped for 22 minutes. After clamp removal, kidneys were observed for restoration of blood flow by the return to original color. The abdomen was closed in one layer. Sham surgery consisted of the same procedure except that clamps were not applied.

### Intratracheal (IT) IL-6 or endotoxin administration

Mice were anesthetized with IP Avertin, positioned against an angled restraining stand and the test material was instilled into the trachea by using a ball tipped needle purchased from Popper and sons (New Hyde Park,NY). For the IT IL-6 experiments, mice received 200 ng or 25 ng of IL-6 (PeproTech #216-16) or 0.1% sterile BSA in a total volume of 50 ul. For IT endotoxin experiments, 80 µg LPS (Sigma Aldrich, Escherichia coli 0111:B4, catalog number L2630) in sterile saline in a total volume of 50 uL was administered (50 µL sterile saline was used for vehicle).

### Intraperitoneal endotoxin administration

10 µg of LPS (Sigma Aldrich, Escherichia coli 0111:B4 catalog number L2630) in 100 µL of normal saline in the lower left abdominal quadrant. This dose triggers an intense systemic proinflammatory response and is commonly used in animal models of sepsis. Sterile saline was used as the vehicle.

### Serum, BAL fluid, and tissue collection

After intraperitoneal (IP) sodium pentobarbital anesthesia, the trachea was dissected and cannulated with a 20 gauge catheter. Lungs were lavaged with 1 mL 1% ethylenediaminetetraacetic acid (EDTA) in PBS two times.

Blood was obtained via cardiac puncture. To ensure uniformity, all samples were processed identically. Blood was allowed to clot at room temperature for 2 hours, then centrifuged at 3000 *rpm* for 10 minutes. Serum was collected and centrifuged a second time at 3000 *rpm* for 1 minute to ensure elimination of red blood cells.

Lungs were snap frozen in liquid nitrogen and stored at −80.

### Serum, lung, and BAL fluid IL-6, CXCL-1, TNF-α and IL-1β and IL-10 measurement

IL-6, CXCL-1, TNF-α, IL-1β, and IL-10. were determined in serum, whole lung homogenates, and BAL fluid by ELISA (R&D Systems, Minneapolis, MN, USA). Lung measurements were corrected for protein and lung tissue was prepared as previously described [11]. The mean minimum detectable dose (range) for each of the cytokines (pg/mL) is as follows: IL-6 1.6 (1.3–1.8); CXCL1: 2.0 (range not reported); TNF-α: 1.88 (0.36–7.21), IL-1β: 2.31 (0.46–4.80), IL-10: 1.97 (0.625–5.22) and this information is included in the methods. The IL-6 ELISA detects both endogenous IL-6 and the recombinant IL-6 that was administered intra-tracheally.

### Lung myeloperoxidase (MPO) activity

Lung MPO activity was determined as previously described [10].

### Blood urea nitrogen and serum creatinine measurement

BUN and serum creatinine were measured using quantitative colorimetric assays (Bioassay systems DICT-500 and DIUR-500) or an ACE clinical autoanalyzer (Alpha Wasserman, West Caldwell, NJ).

### BAL fluid cell composition determination

The BAL fluid cell composition was determined by manual counting of stained BAL fluid cells and confirmed by flow cytometry of BAL fluid cells. For the *manual counting method* the BAL fluid cell pellet was resuspended in 1 mL PBS. 10 µl of the suspension was placed on a hemacytometer and total cell count was determined. Additionally, 100 µl of the suspension was centrifuged with a Cytofuge (IRIS International) at 300 rpm onto microscope slides. Cells were fixed and stained using a histological stain similar to Wright-Giemsa stain utilizing Protocol HEMA 3 stain set according to the manufacturer's directions. Neutrophils were counted and the percent of neutrophils relative to the total number of cells was determined. BAL fluid neutrophil counts were confirmed by flow cytometry (data not shown) using a previously described method [27].

### Statistical analysis

All values are expressed as mean ± standard error (SE). Non-parametric t-tests were performed for experiments with two groups. For experiments with greater than 2 groups, one way ANOVA was performed. For t-tests, groups with significant variance were subjected to Welch's correction. A *P* value of ≤0.05 was considered statistically significant.

## Results

### Bronchoalveolar (BAL) fluid IL-6 in acute kidney injury (AKI), intraperitoneal (IP) endotoxin and intratracheal (IT) endotoxin

**Figure 1:**
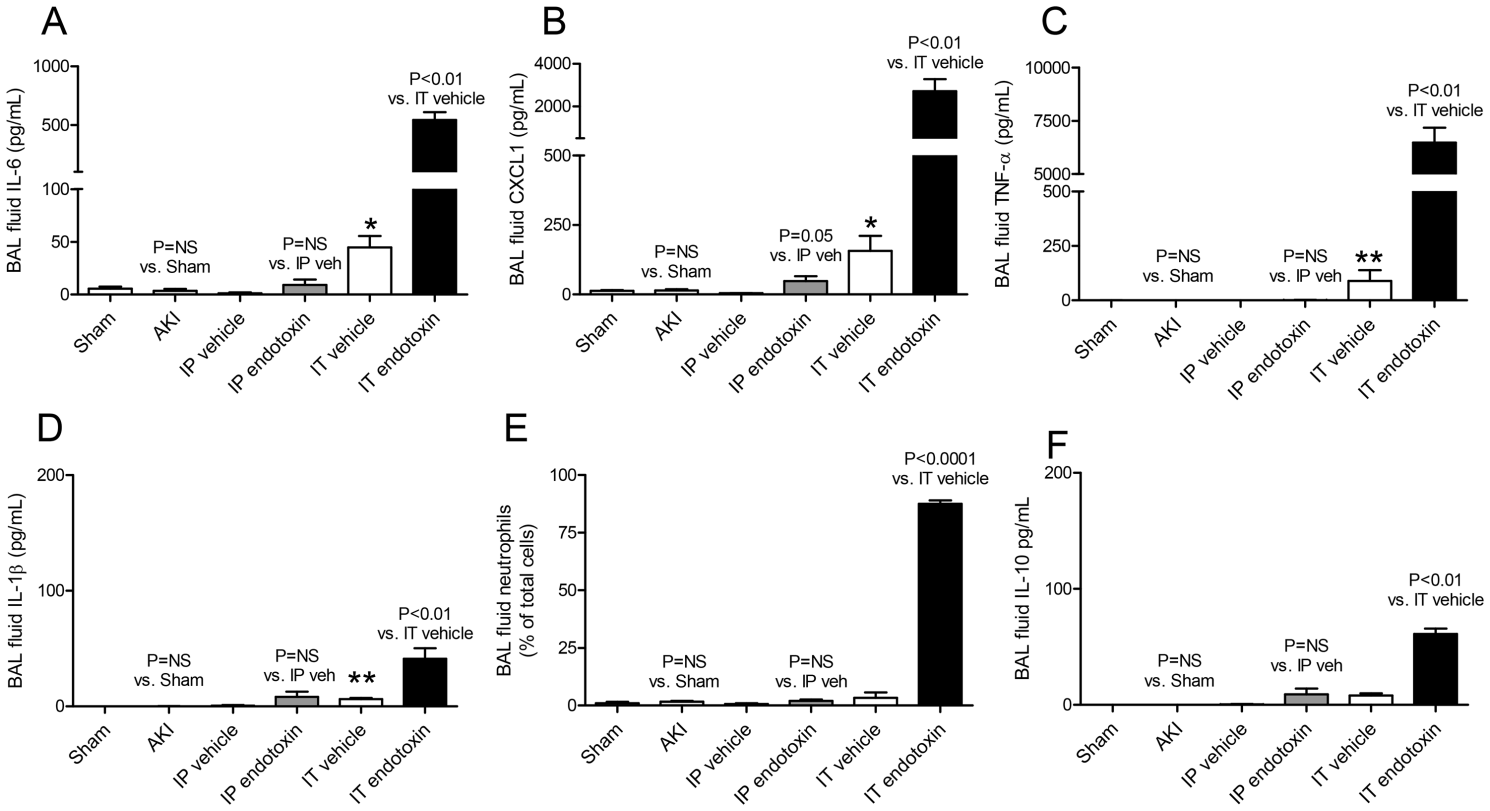
Bronchoalveolar (BAL) fluid proinflammatory cytokines, neutrophils, and IL-10 after acute kidney injury (AKI), intraperitoneal (IP) endotoxin, or intratracheal (IT) endotoxin. BAL fluid proinflammatory cytokines (A) IL-6, (B) CXCL1, (C) TNF-α, (D) IL-1β; and (E) neutrophils; and (F) IL-10 (an anti-inflammatory cytokine) were determined 4 hours after AKI (indirect lung injury), IP endotoxin (indirect lung injury), or IT endotoxin (direct lung injury) (n = 3–7). *P<0.01 versus IP vehicle and P = NS versus Sham; **P<0.01 versus IP vehicle and P<0.01 versus Sham.

To assess the role of intra-alveolar IL-6 in AKI-mediated lung injury, we first measured BAL fluid IL-6 four hours after AKI versus sham operation. As shown in Figure 1, BAL fluid IL-6 was not increased after AKI versus sham operation. Since AKI is a form of indirect lung injury, we questioned whether BAL fluid IL-6 might increase in direct, but not indirect, causes of lung injury. Therefore, we examined BAL fluid IL-6 after IP endotoxin administration (indirect lung injury) and IT endotoxin administration (direct lung injury).

As shown in Figure 1, BAL fluid IL-6 was not increased in IP endotoxin administration but was dramatically increased in IT endotoxin administration. Thus, BAL fluid IL-6 is increased in direct (IT endotoxin) but not indirect lung injury (AKI, IP endotoxin).

Interestingly, IT vehicle (the control group for IT endotoxin) increased BAL fluid IL-6 which was significantly increased versus sham operation and IP endotoxin. Thus, IT vehicle administration itself is proinflammatory as judged by increased BAL fluid IL-6.

### BAL fluid CXCL1, TNF-α, and IL-1β in AKI, IP endotoxin, and IT endotoxin

To determine if other intra-alveolar cytokines might be important in the pathogenesis of AKI-mediated lung injury, the BAL fluid cytokines CXCL1, TNF-α, and IL-1β were determined four hours after AKI versus sham operation. As shown in Figure 1, BAL fluid CXCL1, TNF-α, and IL-1β were not increased in AKI versus sham operation.

To determine if these cytokines, like IL-6, were increased in only direct, but not indirect, lung injury BAL fluid CXCL1, TNF-α, and IL-1β were determined four hours after IP endotoxin or IT endotoxin. As shown in Figure 1, BAL fluid TNF-α, and IL-1β were not increased in IP endotoxin although BAL fluid CXCL1 was minimally increased versus IP vehicle. In contrast, BAL fluid IL-6, CXCL1, TNF-α, and IL-1β were all increased after IT endotoxin versus IT vehicle.

### BAL fluid neutrophils in AKI, IP endotoxin, and IT endotoxin

Since proinflammatory cytokines facilitate the recruitment of neutrophils, we determined whether the presence of BAL fluid cytokines correlated with an influx of neutrophils into the alveolar space. Thus, BAL fluid neutrophils were determined 4 hours after AKI, IP endotoxin, or IT endotoxin. Consistent with the BAL fluid cytokine values, BAL fluid neutrophils were not increased after AKI or IP LPS, but were dramatically increased after IT LPS, as shown in Figure 1.

Thus, direct lung injury (IT endotoxin) is characterized by marked inflammation in the alveolar space as judged by BAL fluid proinflammatory cytokines while indirect lung injury from either AKI or IP endotoxin is characterized by absent or very minimal inflammation in the alveolar space.

### BAL fluid IL-10 in AKI, IP endotoxin, and IT endotoxin

To further characterize the cytokine response in the alveolar space in indirect and direct lung injury, we examined the BAL fluid for IL-10. IL-10 is the prototypical anti-inflammatory cytokine which is critical for the counter inflammatory response that contains and inhibits proinflammatory cytokine production after injury. As shown in Figure 1, BAL fluid IL-10 was not increased in indirect lung injury (AKI or IP endotoxin), but was increased after direct lung injury (IT endotoxin). Thus, the counter inflammatory response is present in the alveolar space by 4 hours post IT endotoxin injection, although proinflammatory effects predominate at this point based on the massive influx of neutrophils into the alveolar space.

### Lung parynchymal inflammation in AKI, IP endotoxin, and IT endotoxin

The hallmark of ALI is chemokine upregulation and lung neutrophil accumulation [28]. We have previously demonstrated that AKI-mediated lung injury is characterized by increased lung CXCL1 (a neutrophil chemokine) and MPO activity (a biochemical marker of activated neutrophils) [10], [11]. Although BAL fluid cytokines were different in the present study, we hypothesized that lung parenchymal inflammation might be similar in indirect and direct causes of lung injury.

**Figure 2:**
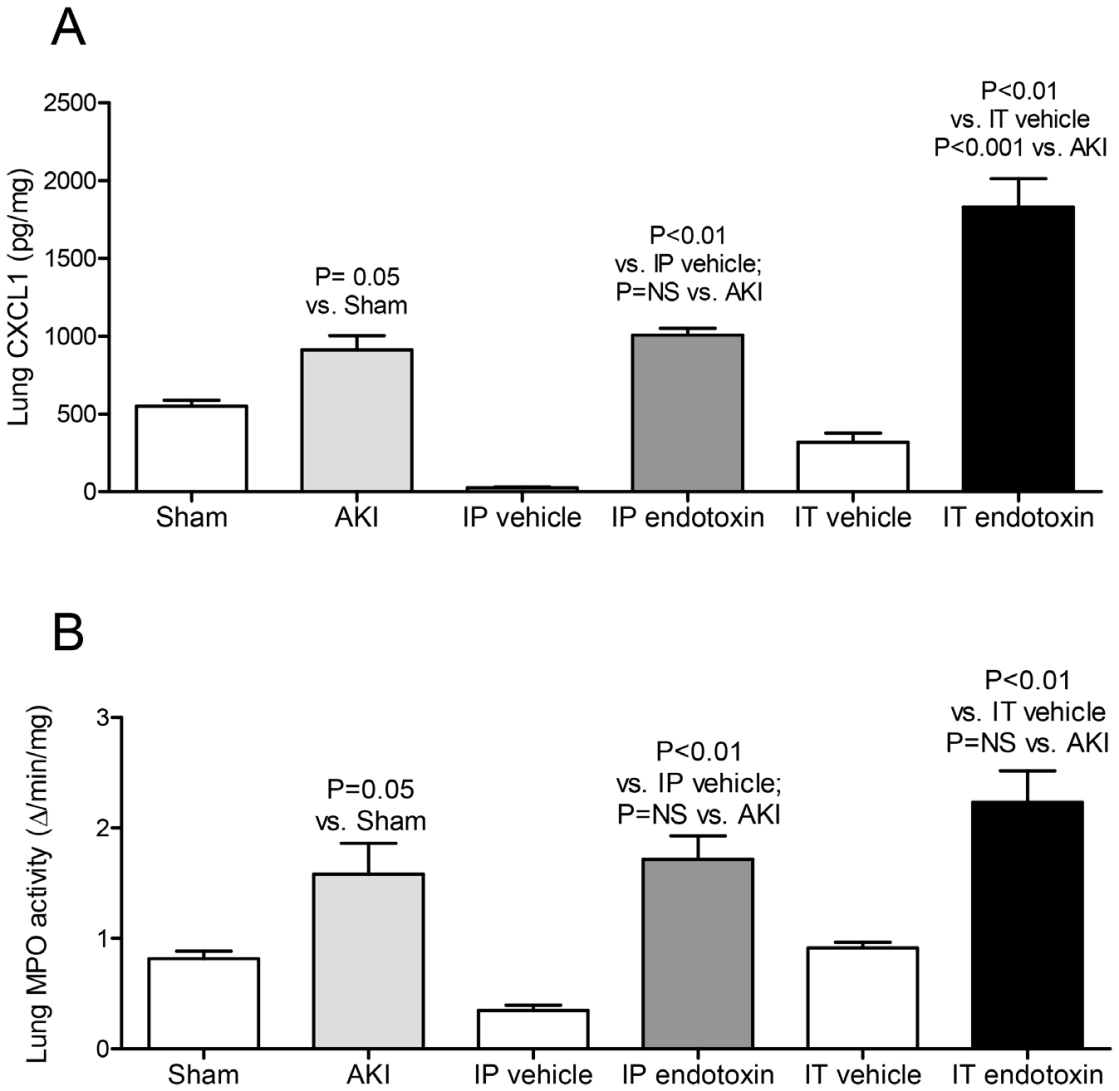
Lung inflammation after acute kidney injury (AKI), intraperitoneal (IP) endotoxin, or intratracheal (IT) endotoxin. Lung (A) CXCL1 protein, and lung (B) MPO activity (a biochemical indicator of lung neutrophils) were measured 4 hours after AKI (indirect lung injury), IP endotoxin (indirect lung injury), or IT endotoxin (direct lung injury) (n = 5–7).

Therefore, lung CXCL1 and lung MPO activity were determined 4 hours after AKI, IP endotoxin, and IT endotoxin. As shown in Figure 2 AKI, IP endotoxin and IT endotoxin were all associated with increased lung CXCL1 and increased lung MPO activity. In fact, lung MPO activity was not significantly different after AKI versus IP endotoxin or IT endotoxin administration.

Thus, lung parenchymal inflammation as judged by lung MPO activity was similar after AKI, IP endotoxin, and IT endotoxin.

### Serum IL-6 in AKI, IP endotoxin, and IT endotoxin

**Figure 3:**
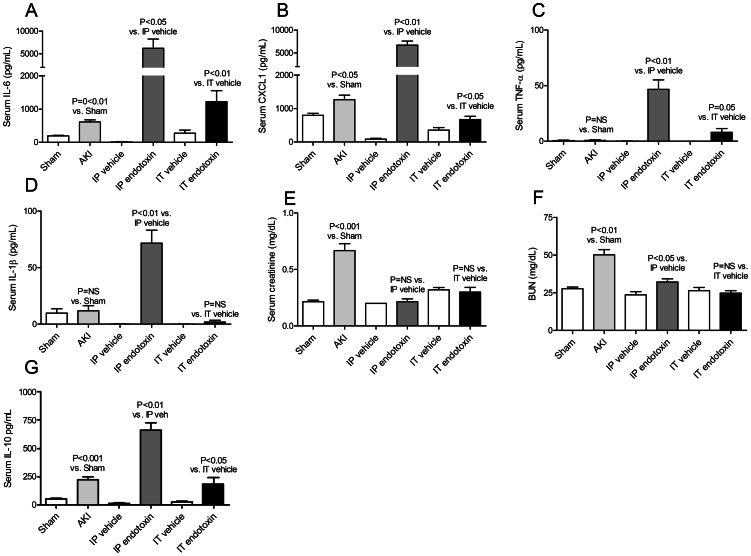
Serum proinflammatory cytokines, renal function, and serum IL-10 after acute kidney injury (AKI), intra-peritoneal (IP) endotoxin, or intratracheal (IT) endotoxin. Serum proinflammatory cytokines (A) IL-6, (B) CXCL1, (C) TNF-α, (D) IL-1β; and renal function by (E) serum creatinine and (F) BUN; and serum IL-10 (an anti-inflammatory cytokine) were measured 4 hours after AKI (indirect lung injury), IP endotoxin (indirect lung injury), and IT endotoxin (direct lung injury)(n = 5–7).

Serum IL-6 is increased in patients with either AKI or ALI and higher levels predict worse outcomes [7], [20], [21], [23], [24]. Since direct lung injury is due to local lung injury, we hypothesized that serum IL-6 would be increased in indirect but not direct lung injury. Therefore, serum IL-6 was measured 4 hours after AKI, IP endotoxin, and IT endotoxin. In contrast to expectations, serum IL-6 was increased in all three forms of lung injury; in fact, serum IL-6 was similar after AKI and IT endotoxin, as shown in Figure 3.

### Serum CXCL1, TNF-α, and IL-1β in AKI, IP endotoxin, and IT endotoxin

To further compare systemic inflammation in indirect and direct lung injury, serum CXCL-1, TNF-α and IL-1β were measured 4 hours after AKI, IP endotoxin, or IT endotoxin. As shown in Figure 3, serum CXCL-1 was increased after AKI; serum CXCL-1, TNF-α and IL-1β were increased after IP endotoxin; and, serum CXCL-1 and TNF-α were increased after IT endotoxin.

Thus, both indirect and direct lung injury are characterized by systemic inflammation as judged by increased levels of the proinflammatory cytokines IL-6 and CXCL1 in the serum; although circulating cytokines were dramatically higher in the IP endotoxin model.

### Serum creatinine and BUN in AKI, IP endotoxin, and IT endotoxin

As shown in Figure 3, both serum creatinine and BUN were increased after AKI, and BUN was increased after IP endotoxin. No change in renal function was observed with IT endotoxin. Thus, renal dysfunction, as judged by increased BUN, is a feature of the IP endotoxin model of indirect lung injury, but not the IT endotoxin model of direct lung injury.

### Serum IL-10 in AKI, IP endotoxin, and IT endotoxin

To examine the systemic counter inflammatory response in indirect and direct lung injury, serum IL-10 was studied. As shown in Figure 3, serum IL-10 was increased after AKI, IP LPS, and IT LPS. Thus, both direct and indirect lung injury are characterized by an increase in serum IL-10 and the initiation of a systemic counter inflammatory response,

### The effect of intratracheal (IT) IL-6 on BAL fluid cytokines, lung inflammation, and circulating cytokines in healthy mice

Although BAL fluid IL-6 was not significantly increased after either AKI or IP endotoxin, it remains possible that intra-alveolar IL-6 may play a role in the pathogenesis of these causes of indirect lung injury. For example, intra-alveolar IL-6 could act locally and be internalized rapidly so as not to be detected in the BAL fluid. Therefore, to obtain further insight into the potential role of intra-alveolar IL-6 in ALI, the effect of intra-alveolar IL-6 was examined in *healthy* mice. Since we have previously demonstrated that *intravenous* administration of IL-6 increases lung CXCL1 and MPO activity in AKI [29], we hypothesized that *intratracheal* administration of IL-6 to healthy mice would also increase lung CXCL1 and MPO activity.

In this experiment, 25 ng or 200 ng of recombinant murine IL-6 or vehicle (0.1% BSA) was administered IT to healthy mice and BAL fluid cytokines (IL-6, CXCL-1, TNF-α and IL-1β), lung inflammation (lung IL-6, CXCL-1, TNF-α, IL-1β, and MPO activity) and serum cytokines (IL-6, CXCL-1, TNF-α and IL-1β) were determined at two hours. Mice without any IT treatment (normal mice) were also examined as a control group.

#### BAL fluid cytokines (IL-6, CXCL-1, TNF-α and IL-1β) after IT IL-6

**Figure 4:**
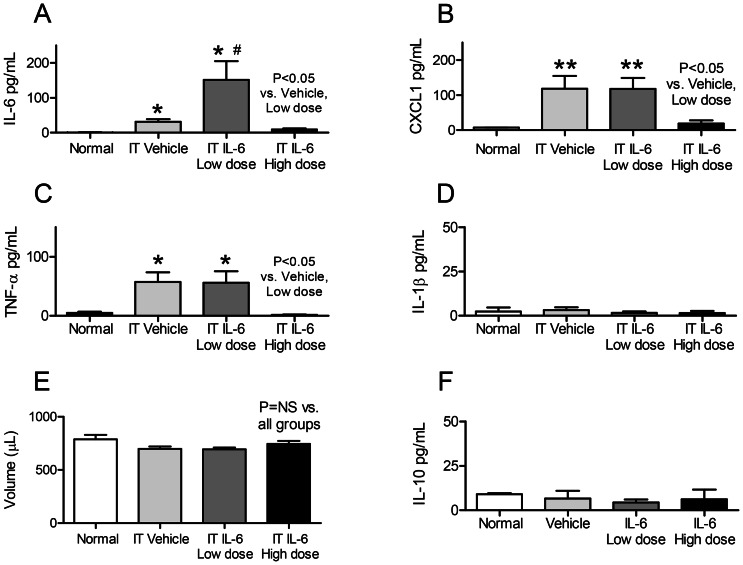
Bronchoalveolar lavage (BAL) fluid proinflammatory cytokines, volume, and IL-10 after intratracheal (IT) IL-6 to healthy mice. BAL fluid proinflammatory cytokines (A) IL-6, (B) CXCL1, (C) TNF-α, (D) IL-1β; and (E) volume; and (F) IL-10 (an anti-inflammatory cytokine) were determined in normal mice (no IT treatment of any kind), and two hours after IT vehicle (0.1% BSA), low dose IT IL-6 (25 ng), and high dose IT IL-6 (200 ng). (n = 3–5 for normal mice; n = 10–20 for vehicle, IL-6 low dose, and IL-6 high dose). * P<0.05 vs. Normal, ** P<0.01 vs. Normal, #P<0.05 vs. Vehicle.

As shown in Figure 4, vehicle-treatment and 25 IL-6-treatment *increased* BAL fluid IL-6, CXCL1, and TNF-α (versus normal mice) while 200 ng IT IL-6 *reduced* BAL fluid IL-6, CXCL1, and TNF-α (versus vehicle-treated and 25 ng IT IL-6). In fact, BAL fluid IL-6, CXCL1, TNF-α, and IL-1β levels were similar in 200 ng IT IL-6 treated and normal mice.

To confirm that the BAL fluid cytokine levels observed were not a result of variable BAL fluid technique among the different groups, BAL fluid volume was evaluated for all four groups examined and, as shown in Figure E, BAL fluid volume was similar in all four groups. Thus, the differences in BAL fluid cytokine levels are not a result of variable BAL fluid technique.

#### Lung inflammation (IL-6, CXCL-1, TNF-a, IL-1β, and MPO activity)

**Figure 5:**
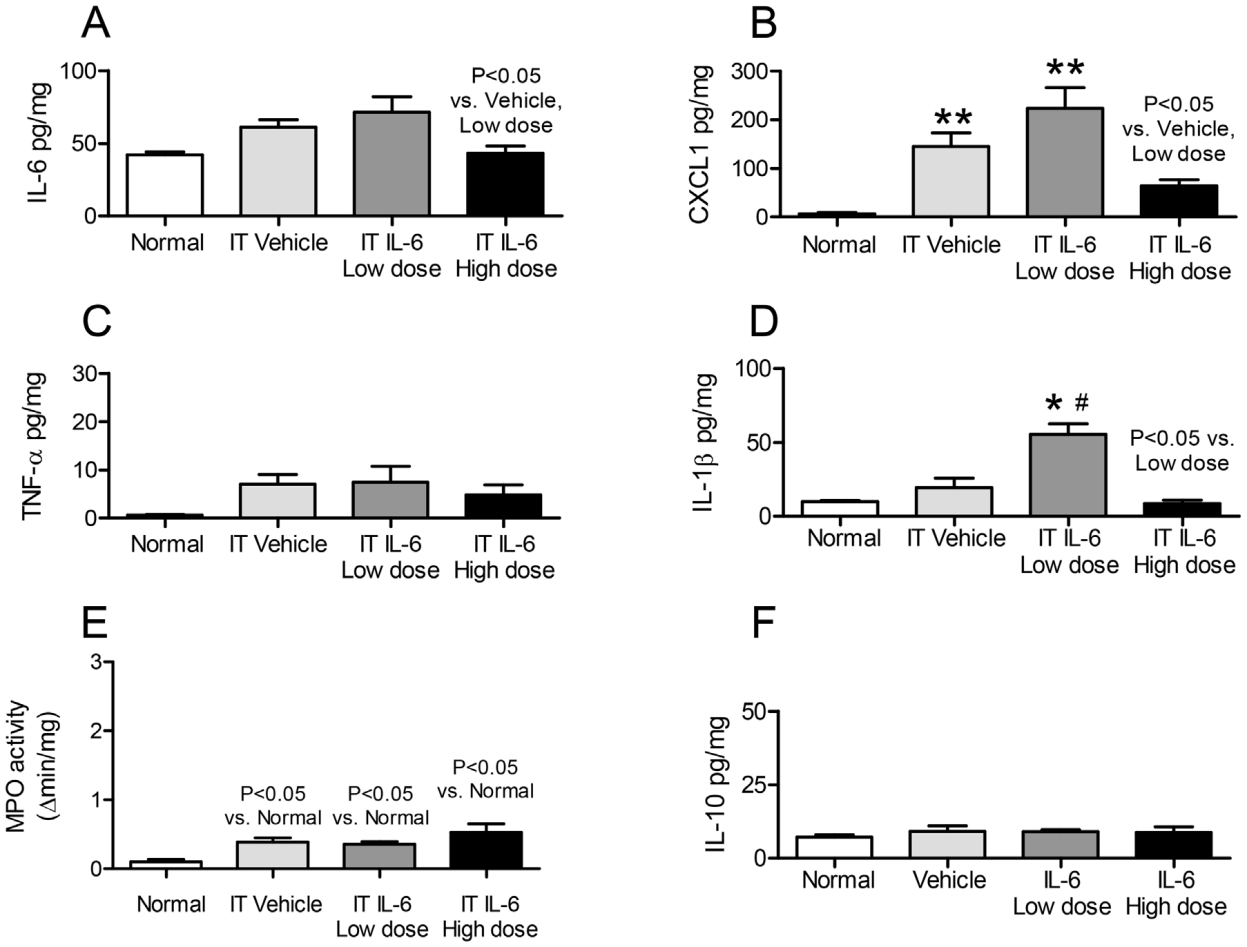
Lung proinflammatory cytokines, lung MPO activity, and lung IL-10 after intratracheal (IT) IL-6 to healthy mice. The proinflammatory cytokines (A) IL-6, (B) CXCL1, (C) TNF-α, (D) IL-1β; and (E) MPO activity; and (F) IL-10 (an anti-inflammatory cytokine) were determined in the lung in normal mice (no IT treatment of any kind), and two hours after IT vehicle (0.1% BSA), low dose IT IL-6 (25 ng), and high dose IT IL-6 (200 ng). (n = 3–5 for normal mice; n = 10–20 for vehicle, IL-6 low dose, and IL-6 high dose). * P<0.05 vs. Normal, ** P<0.01 vs. Normal, #P<0.05 vs. Vehicle.

As shown in Figure 5, IT vehicle increased lung CXCL1 and 25 ng IT IL-6 increased lung CXCL1 and IL-1β versus normal mice. In contrast, 200 ng IT IL-6 *reduced* lung cytokines: lung CXCL1, and IL-1β were *decreased* versus 25 ng IT IL-6 or IT vehicle, and lung IL-6 was *decreased* versus 25 ng IT IL-6. Lung MPO activity was similarly increased after IT vehicle, 25 ng IT IL-6, and 200 ng IT IL-6 versus normal.

#### Serum cytokines

**Figure 6:**
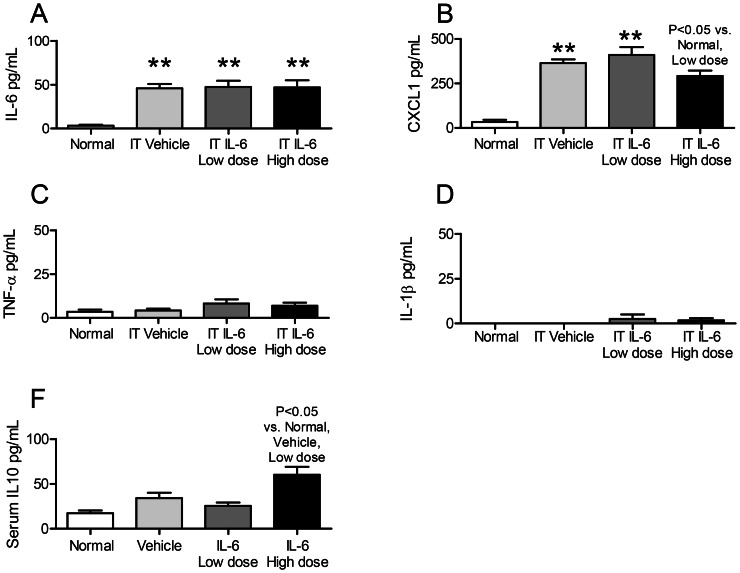
Serum proinflammatory cytokines and serum IL-10 after intratracheal (IT) IL-6 to healthy mice. Serum proinflammatory cytokines (A) IL-6, (B) CXCL1, (C) TNF-α, and (D) IL-1β; and (F) IL-10 (an anti-inflammatory cytokine) were determined in normal mice (no IT treatment of any kind), and two hours after IT vehicle (0.1% BSA), low dose IT IL-6 (25 ng), and high dose IT IL-6 (200 ng). (n = 3–5 for Normal mice; n = 10–20 for Vehicle, IL-6 Low Dose, and IL-6 High Dose). * P<0.05 vs. Normal, ** P<0.01 vs. Normal, #P<0.05 vs. Vehicle.

As shown in Figure 6, IT vehicle and 25 ng IT IL-6 had similar effects on serum cytokines and increased serum IL-6 and CXCL-1 versus normal mice. With 200 ng IT IL-6 treatment, serum IL-6 and CXCL1 were also increased versus normal mice, however, serum CXCL1 was significantly *decreased* versus 25 ng IT IL-6.

#### BAL fluid, lung, and serum IL-10

Because we noted that high dose IT IL-6 had an anti-inflammatory effect, we examined BAL fluid, lung, and serum IL-10 to determine whether the anti-inflammatory effect of high dose IT IL-6 might be due to induction of the anti-inflammatory cytokine IL-10. Neither BAL fluid IL-10 nor lung IL-10 were increased with high dose IT IL-6 (Figures 4 and 5, respectively). Interestingly, serum IL-10 was significantly increased versus normal, vehicle-treated, and low dose IL-6 treated (Figure 6).

In summary, these data demonstrate that IT vehicle treatment in healthy mice is proinflammatory as judged by increased BAL fluid cytokines (IL-6, CXCL1, TNF-α), increased lung inflammation (CXCL1, MPO activity), and increased serum cytokines (IL-6, CXCL1). Low dose (25 ng) IT IL-6 did not have an additional effect on BAL fluid CXCL1 or TNF-α, lung inflammation (CXCL1, MPO activity), or serum cytokines (IL-6, CXCL1) as compared to IT vehicle. In contrast, high dose IT IL-6 resulted in marked anti-inflammatory effects as judged by reduced BAL fluid cytokines (IL-6, CXCL1, TNF-α), lung inflammation (IL-6, CXCL1, IL-1β), and serum CXCL1. The anti-inflammatory effect of high dose IT IL-6 appears to be unrelated to IL-10 induction in the lung or alveolar space, but may be related to a systemic increase in IL-10 as serum IL-10 was significantly increased with high dose IT IL-6.

### Effect of IT IL-6 in AKI, IP endotoxin, and IT endotoxin

Since the previous experiment suggested that 200 ng of IT IL-6 had anti-inflammatory effects in the lung, the therapeutic potential of IT IL-6 was tested in AKI, IP endotoxin, and IT endotoxin. We expected IT IL-6 to reduce lung inflammation in these models of indirect and direct lung injury. In this experiment, 200 ng of IT IL-6 or vehicle (IT 0.1% BSA) was administered 30 minutes prior to AKI induction, IP endotoxin administration, or IT endotoxin administration and BAL fluid cytokines (IL-6, CXCL-1, TNF-α and IL-1β), lung inflammation (lung IL-6, CXCL-1, TNF-α, IL-1β, and MPO activity) and serum cytokines (IL-6, CXCL-1, TNF-α and IL-1β) were determined 4 hours post-procedure.

#### BAL fluid cytokines after IT vehicle administration in AKI versus IP endotoxin and IT endotoxin

**Figure 7:**
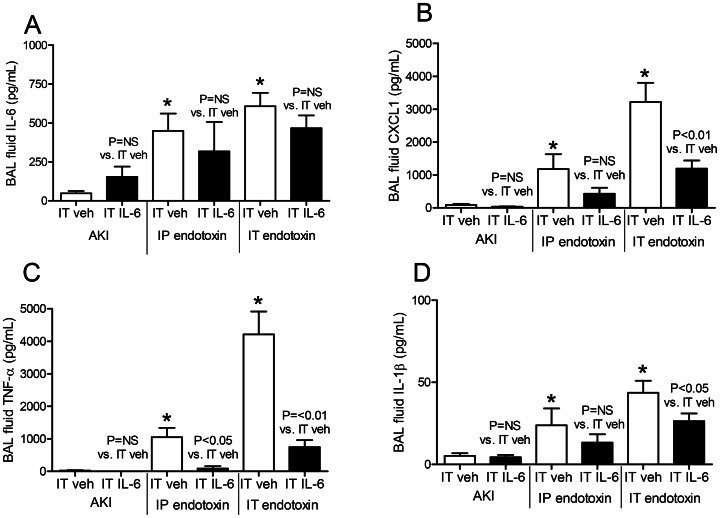
Bronchoalveolar (BAL) fluid proinflammatory cytokines after intratracheal (IT) IL-6 treatment in acute kidney injury (AKI), intraperitoneal (IP) endotoxin, and IT endotoxin. The proinflammatory cytokines (A) IL-6, (B) CXCL1, (C) TNF-α, and (D) IL-1β were measured in BAL fluid 4 hours after AKI (indirect lung injury), IP endotoxin (indirect lung injury), or IT endotoxin (direct lung injury) in mice treated with 200 ng of IT IL-6 or IT vehicle (veh) (0.1% BSA) 30 minutes prior to injury induction (n = 5–7). *P<0.01 versus IT vehicle plus AKI).

As noted above, IT vehicle increased BAL fluid cytokines. Notably, however, in this experiment, IT vehicle did not increase BAL fluid cytokines in mice with AKI; BAL fluid IL-6, CXCL1, TNF- α, and IL-1β were all significantly increased in IT vehicle plus IP endotoxin, or IT vehicle plus IT endotoxin, versus IT vehicle plus AKI (Figure 7). These data suggest that the alveolar space in AKI is hypo-responsive to a proinflammatory stimulus.

#### BAL fluid cytokines after IT IL-6 administration in AKI, IP endotoxin, IT endotoxin

As shown in Figure 7, IT IL-6 had no effect on BAL fluid cytokines after AKI. In IP endotoxin, only BAL fluid TNF-α was reduced in IT IL-6-treated. In IT endotoxin, BAL fluid CXCL1, TNF-α and IL-1β were all *reduced* in IT IL-6 treated. BAL fluid neutrophils were also reduced with IT IL-6 treatment in IT endotoxin; BAL fluid neutrophils (% total cells) were 91±1 in IT endotoxin+vehicle and were 75±7 in IT endotoxin+IT IL-6 (n = 4, P = 0.05).

#### Lung inflammation (lung CXCL-1, lung MPO activity and lung neutrophils)

**Figure 8:**
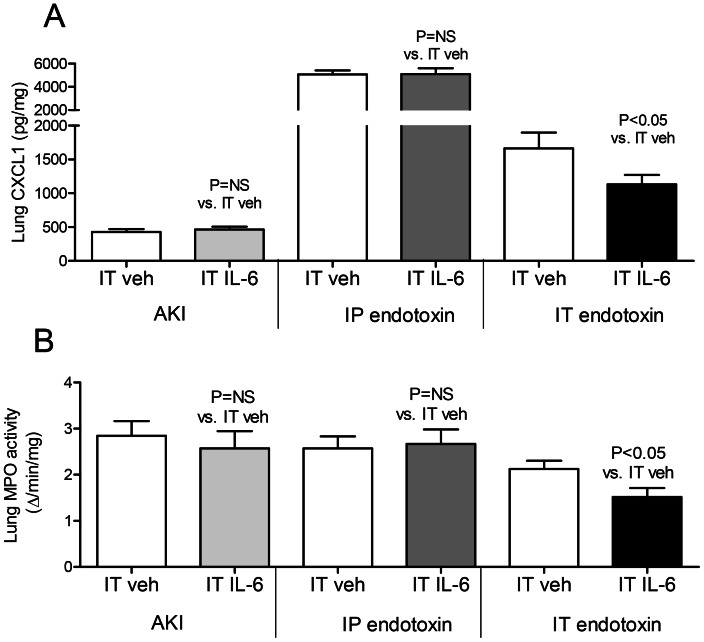
Lung inflammation after intratracheal (IT) IL-6 treatment in acute kidney injury (AKI), intraperitoneal (IP) endotoxin, and IT endotoxin. (A) Lung CXCL1 (a neutrophil chemokine), and (B) lung MPO activity (a biochemical indicator of lung neutrophils) were measured 4 hours after AKI (indirect lung injury), IP endotoxin (indirect lung injury), and IT endotoxin (direct lung injury) in mice treated with 200 ng of IT IL-6 or IT vehicle (veh) (0.1% BSA) 30 minutes prior to injury (n = 5–7).

As shown in Figure 8, lung CXCL-1 and lung MPO activity were not affected by IT IL-6 treatment in either AKI or IP endotoxin. In contrast, IT IL-6 effectively reduced lung CXCL1 and MPO activity in IT endotoxin. Thus lung inflammation is decreased with IT IL-6 in direct lung injury while no effect is seen in indirect lung injury.

#### Serum cytokines (IL-6, CXCL-1, TNF-α and IL-1β)

**Figure 9:**
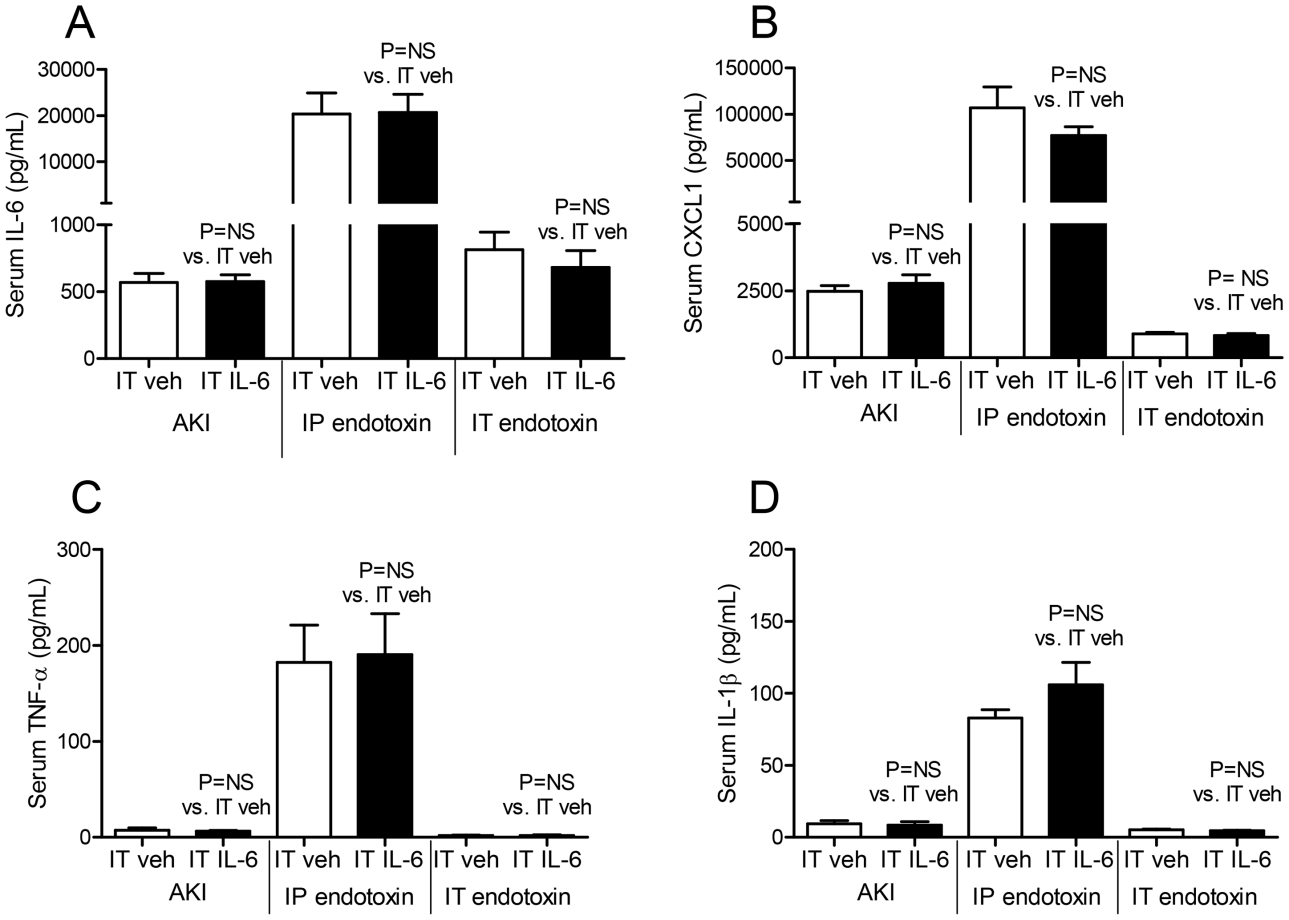
Serum proinflammatory cytokines after intratracheal (IT) IL-6 treatment in acute kidney injury (AKI), intraperitoneal (IP) endotoxin, and IT endotoxin. The proinflammatory cytokines (A) IL-6, (B) CXCL1, (C) TNF-α, and (D) IL-1β were measured in the serum 4 hours after AKI (indirect lung injury), IP endotoxin (indirect lung injury), and IT endotoxin (direct lung injury) in mice treated with 200 ng of IT IL-6 or IT vehicle (veh) (0.1% BSA) 30 minutes prior to injury (n = 5–7).

IT IL-6 had no effect on any of the serum cytokines measured in AKI, IP endotoxin, or IT endotoxin versus IT vehicle treatment (Figure 9).

#### Serum creatinine and BUN

IT IL-6 had no effect on serum creatinine or BUN in AKI, IP endotoxin, or IT endotoxin versus IT vehicle treatment (data not shown). Thus no effect is seen on systemic inflammation or renal function with IT IL-6 in direct or indirect lung injury.

*In summary*, these data demonstrate that pre-treatment with IT IL-6 decreases alveolar and lung inflammation, but not systemic inflammation, in direct lung injury induced by IT endotoxin and has no effect on indirect lung injury induced by either AKI or IP endotoxin.

### Effect of IT IL-6 on IL-10

**Figure 10:**
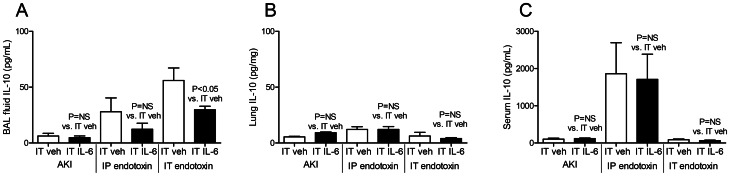
Bronchoalveolar (BAL) fluid, lung, and serum IL-10 after intratracheal (IT) IL-6 treatment in acute kidney injury (AKI), intraperitoneal (IP) endotoxin, and IT endotoxin. The anti-inflammatory cytokine IL-10 was determined in the (A) BAL fluid, (B) lung, and (C) serum 4 hours after AKI (indirect lung injury), IP endotoxin (indirect lung injury), and IT endotoxin (direct lung injury) in mice treated with 200 ng of IT IL-6 or IT vehicle (veh) (0.1% BSA) 30 minutes prior to injury (n = 5–7).

To investigate the mechanism of the anti-inflammatory effect of IT IL-6 in direct lung injury we studied the anti-inflammatory cytokine IL-10 (Figure 10). In mice with AKI or IP endotoxin, IT IL-6 had no effect on BAL fluid, lung, or serum IL-10. Strangely, BAL fluid IL-10 was *reduced* with IT-IL-6 in IT endotoxin, although lung and serum IL-10 were unchanged. Thus, IT IL-6 does not exert an anti-inflammatory effect in direct lung injury (IT endotoxin) by increasing the production of IL-10.

### Effect of IT IL-6 in IT endotoxin when administered after injury

To determine the therapeutic potential of IT IL-6 in direct lung injury, 200 ng of IT IL-6 or vehicle was administered 30 minutes *after* IT endotoxin injection and BAL fluid cytokines, lung inflammation, and serum cytokines were determined; all endpoints were similar in IT IL-6 treated versus IT vehicle treated. Specifically, BAL fluid IL-6, CXCL1, TNF-α, and IL-1β, lung MPO activity, lung CXCL1, BAL fluid neutrophils, and serum IL-6, CXCL1, TNF-α, and IL-1β were not statistically different between IT vehicle and IT IL-6 (n = 7) (data not shown). Thus, IT IL-6 does not improve lung inflammation when administered 30 minutes *after* the onset of direct lung injury via IT endotoxin.

## Discussion

Acute respiratory distress syndrome (ARDS) occurs in approximately 200,000 patients in the United States per year and is a major cause of respiratory failure [30]. ARDS is defined by the development of hypoxemia and noncardiogenic pulmonary edema [31]. This simple definition, without regard to etiology, has been instrumental in the recruitment and study of large numbers of patients with ARDS which has led to a dramatic reduction in the mortality from 40% to 20% in the past 15 years [32], [33] largely due to clinical trials of ventilator management (low versus high tidal volume), protocolized fluid management, and infection control measures. Despite this success, it is now widely thought that further improvement in ARDS outcomes will require distinct treatments that depend on ARDS etiology [34]. Indeed, the failure of drug interventions in ARDS likely rests with the heterogeneity of ARDS etiologies in clinical trials. Furthermore, more basic research into the pathogenesis of different etiologies of acute lung injury has been recommended [34].

Despite the now recognized importance of clarifying the pathogenesis of different causes of ARDS, few studies have compared the development of injury of different etiologies of ARDS, and even fewer have compared the effect of a specific treatment in different causes of ARDS. In the present study, we 1) compared the features of three different animal models of acute lung inflammation, and 2) compared the response of three different causes of acute lung inflammation to a therapeutic intervention. Specifically, we examined AKI-mediated lung injury, IP endotoxin induced lung injury, and IT endotoxin induced lung injury and their response to IT IL-6.

The simplest classification of ARDS with respect to etiology is whether it is indirect or direct. Therefore, to gain insight into the pathogenesis of indirect versus direct lung injury, we first compared the inflammatory response in three compartments: alveolar space, lung and systemic (serum) in indirect (AKI, IP endotoxin) and direct (IT endotoxin) lung injury. Remarkably, lung inflammation was similar, thus allowing accurate comparison regarding the initiating factors of lung inflammation in these models. Additionally, serum IL-6 and CXCL1 were both increased in the three models of acute lung injury; however, major differences were observed in the alveolar space. Direct lung injury (IT endotoxin) was characterized by marked inflammation in the alveolar space as judged by BAL fluid proinflammatory cytokines while indirect lung injury was characterized by absent (AKI) or very minimal (IP endotoxin) inflammation in the alveolar space.

It is widely accepted that alveolar macrophage activation and subsequent proinflammatory cytokine/chemokine production plays a central role in the pathogenesis of ARDS [35], [36]. The lack of meaningful BAL fluid cytokine production early in AKI and IP endotoxin suggests that alveolar cytokine production may not be a major factor in the *initiation* of lung injury in indirect lung injury and that systemic factors are more important. We studied the early events in these models of lung inflammation; thus, it is possible that BAL fluid or serum cytokines may increase later and be involved in the pathogenesis of lung inflammation later in the course of disease. Since lung inflammation was similar four hours post procedure, our data suggest that circulating factors initiate lung inflammation early in indirect causes of lung inflammation while intra-alveolar factors initiate lung inflammation early after direct lung injury.

IL-6 can exert ***either*** pro or anti-inflammatory effects depending on the type of injury and the organ or cell in which it is studied [37]; the conditions that lead to either a pro or anti-inflammatory response have yet to be adequately explained. The role of IL-6 in lung injury, in particular, has led to conflicting results; for example, IL-6 deficient mice exposed to aerosolized endotoxin had *worse* lung inflammation [38], while IL-6 deficient mice with AKI [39] or hemorrhagic shock [40], [41] had *reduced* lung inflammation. These data suggests that IL-6 may be protective in direct lung injury (IT endotoxin), while pathogenic in indirect lung injury (AKI, hemorrhagic shock). In fact, we have previously demonstrated that intravenous injection of IL-6 to IL-6 deficient mice with AKI increases lung CXCL1 and MPO activity demonstrating that *circulating* IL-6 has inflammatory effects. In contrast, the effect of IL-6 in the alveolar space in AKI-mediated lung injury had not been examined. Our data in the present study suggest that alveolar IL-6 has no role in the pathogenesis nor protection against lung inflammation in AKI given that BAL fluid IL-6 was not increased in AKI, and administration of IT IL-6 had no effect on lung inflammation in AKI. Similarly, BAL fluid IL-6 was not increased after IP endotoxin and IT IL-6 had no effect on lung inflammation.

In contrast to indirect lung injury from AKI and IP endotoxin, a dramatic increase in BAL fluid cytokines was noted with IT endotoxin and IT IL-6 did protect against lung inflammation. It is important to note that IT IL-6 has been examined previously in other models of direct lung injury and was also found to have a protective, anti-inflammatory effect [42], [43], [44]; previous studies have demonstrated a reduction in TNF-α production by IL-6 [45], which was also observed in the present study. To our knowledge, IT IL-6 had not previously been examined in indirect models of ALI. Taken together, a pattern of effect of IL-6 in lung injury is emerging demonstrating that alveolar IL-6 plays a protective, anti-inflammatory role in direct lung injury, while having essentially no role in the protection or pathogenesis in indirect lung injury.

It is well known that fever is a complication from bronchoalveolar lavage which is due to proinflammatory cytokine production by alveolar macrophages resulting in increased bronchoalveolar fluid and serum cytokines [46]. This effect was also observed in the present study where IT vehicle resulted in an increase in BAL fluid and serum cytokines which was largely prevented with the administration of IT IL-6; these data further support the notion of IL-6 having an ant-inflammatory effect in the alveolar space. It is interesting and important to note, that, compared to healthy mice or mice treated with IP endotoxin or IT endotoxin, IT vehicle did not increase BAL fluid cytokines in AKI. These data suggest that alveolar compartment in AKI is *hypo*responsive to an inflammatory stimulus. This interpretation is consistent with the observation that patients with AKI are prone to pneumonia and sepsis [47] and that experimentally, mice with AKI have increased susceptibility to bacterial pneumonia [48]. Further exploration of the hypo-immune state in the alveolar compartment might yield important information regarding lung infection risk in patients with AKI.

In summary, the data in the present study advance the understanding of the pathogenesis of lung injury from direct and indirect causes. Our data support the notion that alveolar inflammation is marked in direct lung injury (IT endotoxin) but not indirect lung injury (AKI, IP endotoxin). Thus, alveolar factors are important in the initiation of lung inflammation after direct, but not indirect, lung injury where circulating factors are more important. Furthermore, our data demonstrate that IL-6 plays an anti-inflammatory role in direct lung injury, but does not play a role in indirect lung injury. The beneficial anti-inflammatory effect of IT IL-6 in IT endotoxin, but not AKI and IP endotoxin, further highlights the role of inflammation in the alveolar space in direct lung injury in contrast to indirect lung injury. Finally, our data suggest that differences in the pathogenesis of lung inflammation between direct and indirect lung injury may affect response to treatments; specifically, that intra-tracheal anti-inflammatory therapies are more likely to have a role in the treatment of direct, but not indirect, causes of lung injury. In conclusion, classifying clinical interventions based on indirect or direct causes of lung injury may be a useful distinction for the basis of clinical trials in ARDS.
